# The Hospital. Nursing Section

**Published:** 1903-08-15

**Authors:** 


### The Hospital.

**Nursing Section.**

Contributions for this Section of "The Hospital" should be addressed to the Editor, "The Hospital" Nursing Section, 28 & 29 Southampton Street, Strand, London, W.C.

No. 881.—Vol. XXXIV. *SATURDAY, AUGUST* 15, 1903.

### Notes on News from the Nursing World.

### A MEMENTO OF QUEEN ALEXANDRA.

In the new offices of the Queen Victoria Jubilee Institute for Nurses, at 120 Victoria Street, Westminster, which consist of two upper floors of the building, there may be seen a memento of the gift which enabled the authorities to effect the removal of their headquarters. It consists of the envelope in which her Majesty conveyed a bank note for £1,000 to the treasurer of the institute. Outside the envelope, which is suitably framed, is written the following :—"£1,000, a gift for offices for the Queen's nurses' staff, now established at St. Katharine's, March 1903, A. R." There will thus be preserved, in permanent form, a little souvenir of the interest which Queen Alexandra takes in the work of the institute.

### THE QUEEN AND THE GALWAY NURSES.

During the visit of the Queen to Galway the two nurses attached to the Galway District Nursing Association, Miss Young and Miss Dowling, and the District Nurse at Oughterard, Miss Bird, were presented to her Majesty, who shook hands with each of them. The Queen in conversation with Miss Jesson, the hon. sec. of the Galway Association, asked how long the branches in association with the Queen Victoria's Jubilee Institute had been at work, and being informed that they had been established for six years, said in reply, "May you go on and prosper in the good work."

### THE ANGLO-AMERICAN NURSING HOME AT ROME.

With reference to the remarks respecting the Anglo-American Nursing Home in Rome which appeared in our issue of August 1st, and our promised inquiry, we regret to learn, on unimpeachable authority, that it is quite true that complaints were made to the committee by the whole of the nurses who worked at the Home during the late season. These complaints were set forth in writing, and various particulars were, we understand, given, showing that the nurses had not been properly treated by the matron. Thus far, no complaint was made against the committee, and dissatisfaction with them arose solely from their failure to recognise the importance of a remonstrance signed by the whole of the nursing staff. One result of the very insufficient notice which they took of the document was that in reply to a letter of inquiry sent by one of the subscribers to the Home, each of the nurses submitted a detailed statement of complaints. This statement is, we are informed, so grave that investigation on the part of the committee has become an urgent necessity. In these circumstances, it is the duty of the subscribers to press for action before the opening of another season. The issue is, after all, in their hands, for if they do not continue to support the institution, a crisis in its existence will become inevitable.

### HONG KONG NAVAL HOSPITAL.

The addition of Miss Macpherson and Miss Inniss to the nursing staff at the Royal Naval Hospital, Hong Kong, gives universal satisfaction in that it supplies a long-felt want, and the Senior Service is to be congratulated upon having initiated a movement that its junior might copy with advantage. Hitherto, in this distant outpost of his Majesty's dominions, the "sick-bay steward" and the Royal Army Medical Corps orderly have done all the nursing required in their respective services. Up to the level of their education and instruction they have performed their duties well enough; but it is satisfactory to learn that "Jack Tar" at least is henceforth to enjoy the refining influence and intelligent interest of women whose instincts and intuitions are of the utmost value and show to best advantage in a hospital ward. In the civil service the newest recruit to the nursing staff, Miss Schaefer, has unfortunately contracted plague. As, however, the attack is of a very mild nature she is being nursed in a private ward of the Government Civil Hospital instead of being removed to Kennedy Town Plague Hospital, and she is progressing satisfactorily.

### A NOTABLE APPOINTMENT.

The appointment of Miss Etta Wise as matron of Cring House, Edinburgh, the department for private patients in connection with the Edinburgh Royal Asylum, is noteworthy. Miss Wise is a certificated hospital nurse, and obtained her general training at the Royal Hospital for Sick Children, Edinburgh and the Royal Infirmary, Perth. She also holds th Diploma of the Medico-Psychological Association fo Proficiency in Mental Nursing, and received he mental training at the Perth District Asylum Murthly, N.B. She has also held the post of matron of Farnham House Private Asylum, near Dublin.' She has thus enjoyed a very complete training, as well as a successful career, and she is held in high esteem for the work she has done in perfecting the organisation of the male department of the Stirling District at Larbert by the extensive employment of female nurses, and of hospital methods. The work of reform and progress in the nursing and care of the insane cannot fail to be stimulated by such an admirable choice as that of Miss Wise.

### THE GREEK HOSPITAL, ALEXANDRIA.

As the authorities of the Greek Hospital at Alexandria frequently advertise for English sisters, we think it necessary to advise nurses who contemplate applying for any appointment in that instituticn to make searching inquiries before they sign an agreement for three years. The terms of the agreement are stringent, and one condition is that if a sister leaves before the end of the period she has to refund the cost of her journey. That this frequently happens appears to be the case, as we are informed that during the last three months three sisters have left the hospital because they could not submit to the treatment to which they were subjected. For example, sisters are required to take charge of 120, and sometimes 160 beds Owing to want of quiet during her sleeping hours one night sister was frequently 24 and even 48 hours without sleep. Both in respect to complaints about over-work and want of courtesy on the part of officials, it is, we are assured, useless to approach the committee. But there is, happily, no necessity for experienced English nurses to accept positions in a foreign hospital where they do not receive the consideration which they are entitled to expect.

### NURSES AS TENEMENT HOUSE INSPECTORS.

One of the latest branches of work undertaken by the New York Board of Health is the inspection of tenement houses by trained nurses. It is stated that the experiment so far has been so successful that it will probably be extended. While we do not see in what respect the duties of such officials in this country would differ from those of sanitary inspectors of both sexes, it is easy to understand that a trained nurse, given the possession of other indispensable qualifications, is specially well fitted to act as an inspector of tenement houses.

### PRIVATE NURSING IN MADRAS.

At a time when the question of private nursing in India is a topic of discussion, it is interesting to learn that, under the auspices of a capable superintendent, the Nursing Home in Mackay's Gardens—the cathedral district of the city—is much appreciated. It is a commodious building with extensive grounds and provision for six to seven patients, men, women, and children. Miss Dent, the manager, was trained at St. Thomas's Hospital, where she was afterwards staff nurse. Her experience in India was gained at the Sassoon Hospital, Poona, and she also held the post of assistant matron at the General Hospital, Madras, for five years. In the two latter appointments she was able to gain an insight into many tropical diseases. She is assisted by a staff of four trained nurses, and the Rev. Canon Brittain, who was for a few weeks a patient in the Home last April, bears testimony in the parish magazine of Coonoor to the practical usefulness of the institution. It is satisfactory to know that the efforts of Miss Dent to improve the position of the nurses at the Government Hospital have been followed up by a successful effort to found an organisation which, while of real value to the sick who seek the shelter of the Home, offers, as vacancies arise, an opening to those who prefer private nursing.

### THE ABUSE OF NURSES' UNIFORMS.

"A Cambridgeshire Nurse" complains in another column that nursemaids wear the uniform of nurses, but the most flagrant example of abusing it occurred the other day at Croydon, where, according to a local paper, the bridesmaids at a wedding, which was solemnised at West Croydon Tabernacle, were in "nurses' uniforms, blue cornflower dresses, white apron and caps." It is observed in the report of the wedding that this attire was "most applicable, the bride having served many years in hospital work." There is no suggestion that the bridesmaids themselves were nurses, and if the hospital work in which the bride was formerly engaged was that of a nurse, the selection of uniform which should be strictly confined to those who have the right to wear it, is all the more to be condemned. It is not stated that the two "groomsmen" were clad in wig and gown; but it would have been no more unseemly for them to have masqueraded as barristers, than it was for the bridesmaids to "get themselves up" as nurses.

### THE TRAINING AT PORTSMOUTH INFIRMARY.

The issue of a syllabus in connection with the examinations of probationer nurses at the Parish of Portsmouth Workhouse Infirmary, gave rise to an odd and unnecessarily long discussion at a meeting of the Portsmouth Board of Guardians. The syllabus is signed by Dr. Charles Knott, who describes himself as Knight of Grace of the Hospital of St. John of Jerusalem, as well as medical superintendent. To this description some of the guardians objected; others, however, having referred to the good work done by the Order of St. John of Jerusalem, maintained that there was no reason why the distinction, having been earned, should not be valued and used. In the end a resolution in favour of revising the circular by omitting the title was defeated. The matter is a small one, anyhow, but the syllabus itself shows that much pains are taken to train the probationers thoroughly. It is only five years since the training school was founded, and in view of the difficulties which the medical superintendent and the matron have had to overcome, it is creditable to them that they are now able to attract an excellent class of candidate. We notice that in the third year advanced lectures on diseases of children, are given, and precautions to observe in nursing such diseases taught; also that, when practicable, third-year probationers are allowed to witness cases in the lying-in room.

### DRAWING ON THE RESERVE FUND.

It is not creditable to the people of Colchester that the committee of the District Nursing Association should be compelled to draw upon their reserve fund in order to pay current expenses. The work of the Association seems to be generally appreciated. During last year the nurses paid upwards of 6,000 visits—1,200 more than in the previous year—and all the speakers at the annual meeting testified to the manner in which their services are appreciated in the town. This being so, the town should rise, or be made to rise, to the occasion, and provide, at all events, sufficient money to meet the necessary expenditure. A nursing organisation which continues to fall back upon its reserve fund as the only means of paying its way, is obviously not in a healthy financial position.

### NEWTON ABBOT INFIRMARY.

Nurses still appear to be unwilling or unable to remain at Newton Abbot Infirmary. The completion of the nurses' home by the Guardians was regarded as likely to induce them to stay, but it has not so far had that effect. At the last meeting of the Board two resignations were accepted. One nurse, who had only been in the infirmary for three weeks, and who was paid £2 14s. 2d. for travelling expenses, asked to be allowed to leave on the 15th, as she had accepted a previous appointment which she applied for before coming to Newton. The Guardians very properly took the view that they were entitled to a full month's notice. The other nurse, who had been at the infirmary for six weeks, tendered her resignation, and gave as the reason that she understood that she was engaged as a nurse and not as a lunatic attendant. The explanation of the workhouse master was that an imbecile became ill and was removed to the infirmary and had to be attended by the nurse. If this is all, the nurse has a poor excuse for leaving.

### AWARDS OF MERIT FOR EFFICIENCY.

Three nurses of the Bradford Children's Hospital have been presented by the Mayor with medals. The first was handed to Nurse Annie Dinsdale, the second to Nurse L. Atkinson, and the third to Nurse Mary Weatherhead. The medals have in the centre the title, "Bradford Children's Hospital, Award of Merit for Efficiency," showing through the enamel. Above this the Bradford Arms are worked in heraldic tints, and below the hospital itself is enamelled in colour. A modelled laurel wreath is effectively introduced, and a diaper ground gives a suitable finish. The bar from which the ribbon hangs bears a red enamelled hospital cross, and is of gold for the first, solid silver for the second, and bronze for the third awards, the ribbons being white, light blue, and gold respectively. The winners will hold the medals for 12 months.

### A SUPERINTENDENT NURSE AT FAULT.

At the last meeting of the Walsingham Board of Guardians, the clerk reported that the new superintendent nurse, who commenced her duties on July 13th, tendered her resignation two days later, giving as a reason for her action that she was misled by the advertisement, which stated that there were about 80 inmates in the home. This, she took to mean, was the number of sick patients for her to look after. On her arrival she found out that she had been mistaken, and as she did not consider there was sufficient work in the house for her and the other nurses, she declined to stay. The Guardians accepted her resignation, but refused to refund the amount of her travelling expenses to Walsingham. Nor can we blame them. The superintendent nurse ought clearly to have informed herself as to the actual number of sick patients before she accepted the appointment. In this instance the Guardians have cause for complaint, inasmuch as they have the trouble, not to speak of probable expense, of making another appointment.

### DISTRICT NURSING IN HUDDERSFIELD.

At the sixth annual meeting of the Huddersfield and District Victoria Sick and Poor Nurses' Associations, the report was read and adopted. One of the most interesting of its features was the intimation that the house-to-house collections realised £112, and that the Huddersfield Board of Guardians had voted with the consent of the Local Government Board a subscription of £21. The number of visits paid by the six nurses was 14,458, and the number of operations attended 114. Though the ordinary reliable income for the year was not quite equal to the ordinary expenditure, the special receipts enabled the Committee to show a substantial balance on the right side, and there appears to be every reason to hope that the flourishing state of the organisation will be maintained.

### A WARNING TO BLACKBURN GUARDIANS.

The Blackburn Board of Guardians have been warned by Mr. H. Jenner-Fust, the Local Government Board Inspector, that the single charge nurse whom they have just appointed at a salary of £25 a year, having previously offered £20 in vain, is not anything like adequate provision, considering the sickness at the infirmary. Mr. Jenner-Fust reminded the Guardians that by not having a full staff of charge nurses, they endangered the proper training of probationers, and added that if the infirmary lost its good name as a training school, some difficulty might be experienced in filling up vacancies for them. We hope that the warning will be taken to heart. A training school without an adequate staff of charge nurses is like an army without sufficient officers, and cannot hope to maintain a reputation worth having.

### WESTHOUGHTON NURSING ASSOCIATION.

It is always particularly satisfactory to hear of a nursing organisation making good progress in a straggling district which is necessarily hard to work. The operations of the Westhoughton Sick Nursing Association are carried on under this condition, as the district covered by a single nurse extends to between five and six thousand acres. Last year Nurse Foster attended 195 cases and paid 3,385 visits. Fortunately, the financial support given to the undertaking is satisfactory, and the committee are hopeful that the time is not far distant when the erection of the contemplated cottage hospital may be taken into serious consideration.

### DISTRICT NURSING AT WATERFORD.

At the annual meeting of the Waterford District Nursing Association, presided over by the Bishop of Waterford and Lismore, it was stated that the number of visits paid by the nurses last year exceeded 11,000. It is only three years since the first of the Jubilee nurses was appointed, and the work has therefore rapidly developed. The Bishop, in commending it to his audience, said that it was one that appealed most strongly to the human heart. The financial position is sound, and there is a credit balance exceeding £29.

### SHORT ITEMS.

The s.s. *Dunera*, which arrived at Southampton on Saturday, had on board Sister J. H. Marks, of Queen Alexandra's Imperial Military Nursing Service, on leave for 65 days.

### The Nursing Outlook.

"From magnanimity, all fear above;
From nobler recompense, above applause,
Which owes to man's short outlook all its charm."

### ON EMPIRE DUTY.

The nurse is ubiquitous nowadays: in every land and every clime she is to be found, and often on the distant posts there are few or none to control her, and she has to be a law unto herself. Therefore the words of Florence Nightingale, addressed last week to the gathering of nurses at Lea Hurst, deserve to go all over the world, and our pages, we know, can carry them far:—

"We hear a good deal nowadays about nursing as a profession, but the question for each nurse is, 'Am I living up to my profession?' The nurse's life is, above all, a moral and practical life, a life not of show, but of practical action. I wish the nurses God-speed in their work, and may each one strive, with the best that is in her, to act up to her profession, and to rise continually to a higher level of thought and practice, character, and dutifulness."

Words for all of us; but more especially for those whose work is in difficult and far-off lands, and to whom the temptation to fall into careless ways is specially strong. A nurse, or a group of nurses, arrive, after a voyage, in a country that is unfamiliar to them, and are met and greeted with pleasure and friendliness by the small white colony in the place. That colony is chiefly male as a rule, and consists of younger sons and those on whom the conventions of England have jarred. They are generally a good-humoured and generous set, not too strict with regard to conduct and clothes, capable of very big virtues and very small vices. To a nurse straight from three years of rigid training the freedom and fellowship is delightful, and the "native" to do all the hard work is a new and toil-dispelling find. That the white man or the white woman is a "boss" who merely commands and never does; that there is only one society which includes all whites, and that there are no rich and no poor in it, but all well to do; offers up an opening for laziness and a closing of the bowels of compassion that is occasionally a pitfall. The young doctors to be found at these stations are not always free from blame, for they are apt to lay more stress on the new nurse's looks than on her professional merits. And the class of patients is so different: if it be a European hospital the patients belong to the one class to whom all whites belong; if it be a native hospital the nurse's duties are chiefly of superintendence.

All these difficulties bring out the best points in a thoroughly good staid woman; she takes up her burden and throws herself into her work with that steady aim towards the maintenance of a high professional ideal that Miss Nightingale insists on. Her hours on and off duty are as rigidly kept as in England; her dress, while adapting itself to the climate, is always strictly nurse-like while on duty, and within the wards no relaxation of professional discipline is ever permitted. And so the respect as well as the admiration of the whites is obtained, and as for the natives they will next door to worship the "Burra Mem-Sahib," the "Big Missus," or whatever she may be called by them in their quaint mixture of languages. Under the rule of such a woman the hospital, large or small, is as clean and antiseptic as in England, the order of the day is as peaceful and as thorough, and the best of nursing is at the service of white or coloured alike.

Now turn to the other side of the picture. A doctor who has lately returned from Johannesburg describes the hospital there as untidy, if not dirty; all the hard work is done by Kaffir boys and the nurses wander about decked in diminutive caps, gold bracelets, rings, and all sorts of unnurselike toilet items. They receive male friends in the nurses' home nearly every evening, and many of these friends are old patients. "There is one part of the hospital which is thoroughly kept and well nursed, and that is the Kaffir part, over which the Roman Catholic sisters reign," he says. Now this is an account of some time ago, and since it was written the late matron of Bradford Infirmary has gone to Johannesburg, and we have every confidence from what we know of Mrs. Magill that she will work a reform in the nursing ideals, and do her best to live up to her profession and to lead others to do the same. But we do not like it ever to be said that the trained nurse is less devoted, less hard-working than is the religious sister.

Different conditions of life and work do not necessarily mean a lowering of the standard; special difficulties should be met and not shirked. "'Tis one thing to be tempted, another thing to fall." Far be it from us to underrate the trials of a nurse who far from her native land and her own folk, has to take up work that is strange amongst a people whose very language is unknown. But the English people have accepted the white man's burden of ruling and colonising far and wide, and in the steps of the soldier follow the steps of the nurse. Her labours are no less necessary for the furthering of the Empire than are those of the soldier and the statesman, and of every Englishwoman as of every man, we expect that she will do her duty. To throw up her post for the first facile offer of marriage in a station where white ladies are almost unknown, to allow her working hours to be disturbed by idle callers, and her wards to be untidy for want of the constant yet kindly supervision needed by natives—are these things compatible with the advice of Florence Nightingale—greatest of nurses and best of Englishwomen?

### Lectures on Medical and Surgical Nursing.

By John Hopkins, F.R.C.S., Medical Superintendent of the Central London Sick Asylum District Asylums, Cleveland Street, W., and Hendon, N.W.

### LECTURE XIV.—DISEASES OF THE SKIN.

Each of the several parts which enter into the structure of the skin may be solely or in a great degree the only one that is diseased. The epidermis, the true skin; the glands, sebaceous or sudorific; the hairs, the hair follicles, the blood-vessels, may be each the principal seat of disease. A great variety of forms of disease are seen in the skin. When the epidermis is mainly affected, if the disease be acute, fluid is poured out into its deeper layers, so that the outer horny layers are thrust up by the effusion. These bags or blisters are called vesicles, if small, and if large they are bullæ. Vesicles are formed also by the blocking of the mouths of sweat glands. This is called an eruption of sudamina. The sweat accumulates and forms little bags of clear fluid under the epidermis, which are seen most often in acute rheumatism.

If the inflammation of the skin be not sufficiently acute to cause a rapid effusion of fluid it may cause the outer layers of epidermis to separate from the deeper ones as scales. This happens in scarlet fever. In erysipelas the inflammation may cause the formation of bullæ, and later on is the cause of peeling. In eczema there is general redness, with swelling of the skin due to the inflammation, and typical vesicles are seen in this disease. If the eczema be chronic there is no blistering, but the skin peels. When the vesicles of acute eczema burst there is discharged a large quantity of fluid similar to that in the vesicles. It is sticky, and soon congeals, forming crusts or scabs. The secretion is a good ground for bacteria to grow in, which in their turn may be quite enough to keep up the eczema. Eczema varies in its characters in the same patient and at one and the same time. Whilst one part is dry and scaling another may be weeping. Quite a different disease is psoriasis, in which the epidermis rapidly grows and is partly separated in thick silvery scales, which lie one on another, and form a thick layer. Psoriasis is a very difficult disease to permanently cure. Patient perseverance with local remedies will remove the disease for a time, but it generally returns.

When the skin is congenitally defective, lacking sebaceous and sweat glands, it is unnaturally dry, and the epidermis tends to scale off, and the surface feels rough. This condition is known as xeroderma. When in an exaggerated form the condition is called ichthyosis, a word which indicates that there are scales on the skin, overlapping like those of a fish. Not only does the epidermis form thick scales, but the papillæ of the skin grow very long and project into the scales, so that when these are pulled off the papillæ are torn across, and the part bleeds. A similar condition to this is seen in cases of hypertrophy of the foot, due to extensive and long-standing ulceration of the leg. Corns are due to a local overgrowth of epidermis excited by friction and pressure. There is a hypertrophy of the papillæ here also. The corn grows in thickness, and pressing upon the true skin beneath causes atrophy of it, and forms a bursa which is apt to inflame and suppurate.

When the true skin is inflamed in its deeper parts where the sweat and sebaceous glands lie, there is generally a tendency for the inflammatory effusions to take place here and there in excess, often round the glands or hair follicles, and the result is that little lumps are felt and seen on the skin. These are called papules, and they may remain as papules throughout their course, or they may go on to the formation of pus in their centres, in which case the pus appears at the top of the papule, as a little mattery head. This is then called a pustule. In small-pox, first a papule forms, then this vesicates, and finally it becomes pustular. A pustule is a small skin abscess. Eczema, in its typical form, produces vesicles only, but it may produce papules and pustules.

The sebaceous glands are frequently the seat of disease. The secretion may be too oily and abundant, giving a greasy appearance to the skin, or it may be too dry, and failing to leave the duct of the gland, dries and hardens in the mouth of the duct; dust and dirt being mixed with it turns the hardened secretion black. The gland thus blocked goes on secreting, and the sebum accumulates and dilates the duct of the gland. This condition is known as acne. If the acne spots inflame there is effusion around them and pus is formed. A little abscess is formed in the sebaceous gland. This may cause permanent disfigurement from pitting. If the mouth of the gland remains blocked and there is no inflammation, the constant accumulation of sebum gives rise to the formation of a bag, which may grow continuously until a cyst as large as a hen's egg or larger is formed. This is known as a sebaceous cyst. The only remedy for a cebaceous cyst is excision.

Parasitic diseases are caused both by vegetable and animal growth. Ringworm, or tinea tonsurans, is due to a fungus that invades the hair. It grows in the substance of the hair and penetrates deeply to its root. By passing over the surface of the skin it extends from one hair to another, and extending in this manner in all directions it forms a ring or circular patch on the scalp, in which the hairs are seen to be short, for they become fragile and break off and split at the end. The object aimed at in applying remedies is to prevent extension and to cure the diseased part. As ringworm is easily communicated from one to another, care has to be taken to prevent those suffering from it coming into contact with the healthy. By covering up the disease the dissemination of the fungus is limited. By shaving the scalp a further step is taken towards the same end, besides enabling the parts to be washed free from dandruff and the remedies to be more thoroughly applied. By epilation, pulling out the hairs by the roots over the diseased patch, the most effectual step has been taken to eradicate the disease.

Tinea versicolor is commonly seen on the bodies of consumptives. It is generally found on those who wear flannel vests and perspire much; and it is confined to the parts covered by the flannel. It is due to the growth of a fungus which forms little scales upon the skin that can with a little perseverance be scratched off. This skin disease is readily cured by the use of soap and water and appropriate local applications, and it should never be seen in any case that has been a week in hospital.

Animal parasites give rise to skin eruptions.

*Scabies*, or itch, is due to an insect which burrows in the deeper layers of the epidermis, and as it progresses, it deposits an egg from time to time in the burrow. It especially is to be found where the skin is soft: in the clefts of the fingers, in the lines formed by the wrinkling of the skin at the bend of the wrists, and wherever the skin is thin and easily penetrated. It causes much itching, and excites inflammation along the burrows, which may lead to the formation of pus. The scratching induced excoriates the skin. If of long standing the whole body may be covered, but it very rarely extends higher than the collar.

Sulphur kills the insect. In applying it care must be taken not to continue the use of it too long, for it is irritant and may excite an eczema that will deceive you into the belief that the disease is not cured. The clothing that a patient has worn and the bedclothes he has slept in need disinfection.

Lice cause eruptions on the body. There are three kinds. The body louse is the largest, and is found in the clothing or in the axilla or beard. It lays its eggs, called nits, on the fibre of the body clothing. Its bite causes irritation and raises a little lump, in the centre of which may be seen a red speck of blood. By scratching the lump may be made to bleed, and the skin may bear evidence of severe scratching by lines of excoriation. In order effectually to get rid of the lice there must be a complete change of clothing, a bath given with the use of carbolic soap, and the clothes taken off must be baked.

Head lice lay their nits in the hair, to which they are firmly glued. Difficulty is often experienced in getting rid of them, and perseverance is necessary. When the hair is full of them, matted together and much eczema of the scalp and nape of the neck has been excited, the only thing to be done is to remove the hair. The greater quantity of nits are found generally in the hair at the back, especially in women. This part may be cut away, leaving sufficient to hide the part that has been denuded. Washing with soap and carbolic acid lotion will dislodge the lice, which lie so close that simple combing will not remove them. The nits may be loosened by the use of some solvent. Acetic acid will do this; but it is generally found that it is a tedious and ineffectual process to comb out the loosened nits, and resort is had to the method of daily washing and combing so long as there remain any nits unhatched.

The third form of lice frequents the hair about the pubes. They are flat and lie close upon the skin clinging to the base of a hair, and they lay their nits upon the hairs. They cause much itching. Shaving and the use of a mercurial ointment is necessary.

The skin is the frequent seat of syphilitic affections. There is probably no form of skin eruption that may not be closely imitated by syphilis.

Lupus is due to the presence of tubercle bacilli in the skin. It is a chronic slowly spreading disease that produces great disfigurement of the face, where it is most commonly seen. Operative measures are necessary to eradicate it, or the use of the $x$ or other rays. In using the ray treatment the object aimed at is to produce a reactionary inflammation. Care has to be taken, however, that too much reaction is not caused, as ray burns are produced that induce sloughing, and the resulting ulcers are very slow to heal.

Rodent ulcer, which is a very chronic slow form of ulceration seen in old people and generally beginning about the eyelids, is treated by $x$-rays also.

In dealing with diseases of the skin, where there is any discharge, crust, raw or ulcerated surface, it is to be remembered that here is a favourable ground upon which bacteria may lodge and grow, and thus perpetuate, intensify, or change the character of the disease. First of all then, the diseased surface must be cleaned and disinfected, and afterwards efficiently protected from dust. When itching is present it is often intense and excites scratching and disturbs the patient's rest. This then must be allayed, and the patient must have proper sleep. If the itching be allowed to persist the patient's scratching will aggravate the condition. Baths, general and local, antiseptic and soothing, are frequently required in skin disease as well as for curative and cleansing purposes.

*Chronic ulcer* of the leg is often difficult to heal completely. Rest in bed, and appropriate dressings will heal it up to a certain point, and then the granulations are seen to begin to melt away at some part, and the healing process ceases. This is often due to the base of the ulcer being fixed to the muscle beneath, so that each time the patient bends his foot it disturbs the granulations. When the foot is fixed by a back splint with a footpiece the ulcer begins to heal again. The contraction that takes place in the scar as the ulcer heals limits the supply of blood to the ulcer. Friction so as to induce redness of the limb is then of use by drawing blood to the part. Skin-grafting will greatly shorten the time of healing of an ulcer, especially if a large one, but the ulcer must be thoroughly clean for the graft to grow.

### Nurses at Lea Hurst.

### FLORENCE NIGHTINGALE'S EARLY HOME.

On Thursday afternoon last week the Mayor of Derby, the Hon. F. Strutt, invited the nurses connected with the Derbyshire Royal Infirmary, the Nursing Institute, the Children's Hospital, the Home of Rest, the Borough Asylum, the Derby Workhouse, the Belper Workhouse, and other institutions, to visit Lea Hurst, the former home of Miss Florence Nightingale. The party consisted of over 70 nurses, and they left Derby by the 1.32 Midland train for Whatstandwell. Upon arriving a portion drove to Lea Hurst, whilst the remainder, headed by the Mayor (who is third cousin to Miss Nightingale), had a most enjoyable walk to the house. There the nurses were most kindly received by Mrs. Shore Nightingale, the present owner of the place. They were taken over the beautiful old house by that lady, who explained many of the things connected with Miss Nightingale's life, which were naturally of great interest to the nurses. There was a magnificent painting of the hospital at Scutari, where Miss Nightingale worked night and day amongst the wounded soldiers, her bedroom, the nursing badge she wore at Scutari, etc. A stroll was then taken in the beautiful garden, after which tea was partaken of in a large tent which had been erected on the lawn. Amongst those present were the Mayor and Mayoress, Mrs. Shore Nightingale, the Revs. H. C. Montford and F. Windley, Dr. McDonald, Dr. Brownlow Smith, Mr. and Mrs. W. G. Carnt (Derbyshire Royal Infirmary), Miss Sparshott (matron of the Infirmary), Mrs. Foster (matron of the Belper Workhouse), Miss Atthill (superintendent of the Home of Rest), Miss Agnes Atthill (superintendent of the Nursing Institute), Sister Trow (the Convalescent Home, Holbrook). Miss Sinclair (of the Borough Asylum), and others. At the conclusion of an excellent tea,

#### Letter from Florence Nightingale.

The Mayor said that before they separated, although they were not going to have any speech-making or votes of thanks, he should like to say a few words. It gave him great pleasure to meet such a gathering as he had the good fortune to see before him. He thought it was rather a pleasant thing that so many of them could be allowed to come together at such a beautiful spot. There was a particular interest in that particular spot inasmuch as they knew it had been the home of Florence Nightingale. It was no longer the home of Florence Nightingale, but still it was the home of one who bore the name of Nightingale, and most people in Derbyshire connected Lea Hurst with the home of the

### NURSES AT LEA HURST—*Continued.*

lady who made herself so illustrious now almost fifty years ago. She set an example not only to the women of England, but to the world, by the way she went into the nursing question and set to work to create almost a new era in the management of hospitals, and more particularly nursing. He had written to Florence Nightingale about their gathering that day, and she had sent the following letter:—

10 South Street, Park Lane, W.
July 28, 1903.

Dear Mr. Strutt,—I am very glad to hear that the nurses of Derby are to spend a day at Lea Hurst. It will be a great pleasure to me to think of them there on that day. Will you express to each and to all of them my warmest wishes for their very highest success, in the best meaning of the word, in the life's work which they have chosen? We hear a good deal nowadays about nursing as a profession; but the question for each nurse is, am I living up to my profession? The nurse's life is above all a moral and practical life—a life not of show, but of faithful action. I wish the nurses God speed in their work, and may each one strive with the best that is in her to act up to her profession, and to rise continually to a higher level of thought and practice, character and dutifulness. My heartiest sympathy and greeting to all.

Yours very sincerely,
Florence Nightingale.

The reading of the letter was followed by considerable applause, and the Mayor continuing, said he thought it had been a great moral privilege and a great pleasure to have such words sent as an encouragement to them in the work they were doing. He trusted that they would all remember the excellent example set by that wonderful lady 50 years ago, and try to follow it. Some of them might think that it was on account of the Crimea that she came to the front, and that if it had not been for the war she would not have come to the fore. He, however, thought there was a little mistake about that. Miss Nightingaie had been brought up in the lap of luxury, living in the best society, and having every comfort a home could give her, but she determined early in life to devote herself to that profession which was admired by all kind-feeling men and women. He thought Mrs. Shore Nightingale would bear him out when he said that Miss Nightingale, long before the Crimea, had commenced nursing. She devoted herself to it, and studied not only in England, but also on the Continent. For years she studied in Germany, where they had numerous improvements in hospitals and their management. It was Florence Nightingale who decided that it was absolutely necessary for the hospitals to have a properly trained staff of nurses, and before the Crimea she set to work to reorganise one hospital in London, which was still doing a great amount of good. It was only because it was known that Florence Nightingale had taken up the question of nursing, and had studied it very carefully, that she was sent to the Crimea. It was found absolutely necessary that some trained lady should go out, and she was the only person in England who could take out a party of trained nurses, who even now would be considered untrained. She achieved a great success in the hospital at Scutari, and that was entirely due to the way in which she had trained herself before. Her health was very much injured by her splendid work out in the Crimea, and ever since that time she had been employing herself almost continually in doing something to aid and improve hospitals—military and otherwise—throughout England. She had left no stone unturned, and he was sure that if they were to go into the matter they would find that she had worked very hard indeed. Everyone would admit that who knew how hard it was to upset the existing order of things, and her name, with others, would always be remembered in connection with the reorganisation of the management of hospitals. It was not possible for them all to do as Miss Nightingale had done, but they could follow her example. They could do good and useful work in their profession, and if they only did their best they would not have spent their lives in vain. He hoped they had all had an enjoyable afternoon, and in conclusion he heartily thanked Mrs. Shore Nightingale for her great kindness in allowing them to look over her house and use her grounds.

Mrs. Shore Nightingale said that it had been a pleasure to have the nurses at her home, and she had much enjoyed their visit. She thanked Mr. Strutt for his kind words about Miss Nightingale, to know whom was to honour in the highest degree. She would be delighted to assist any who wished to follow in the steps of Florence Nightingale.

Mr. W. G. Carnt proposed a vote of thanks to the Mayor for his kind hospitality, which they all appreciated, and also to Mrs. Shore Nightingale for her kindness. This was agreed to with applause.

The party then had a walk in the district and returned to Derby by the 7.19 train, all having spent an enjoyable and almost unique day. Mr. Carnt, on behalf of the party, sent a telegram to Miss Nightingale thanking her for her kind letter.

### Appointments.

Braintree Workhouse Infirmary.—Miss Rosa Templeman has been appointed superintendent nurse. She was trained at Wandsworth and Clapham Infirmary, and has since been doing private nursing. She has also been charge nurse at Park Hospital, Lewisham, charge nurse at the Union Hospital, Cardiff, and day midwife at Lambeth Workhouse, S.E.

Cring House, Edinburgh.—Miss Etta Wise has been appointed matron. She was trained at the Royal Hospital for Sick Children, Edinburgh, the Royal Infirmary, Perth and the Perth District Asylum. She has also held the post of matron of Farnham House Private Asylum, near Dublin, and matron of the male department of the Stirling District Asylum, Larbert, N.B.

General Hospital, Tunbridge Wells.—Miss Ada M. Smith has been appointed matron. She was trained at Charing Cross Hospital, London, where she has since been sister for five and a half years and temporary matron for six months.

Kelso Infectious Diseases Hospital.—Miss Edith Edeson has been appointed matron. She was trained at the City Hospital, Birmingham, and has since been nurse at the Chester General Infirmary, and ward sister and deputy-matron at the City Hospital East, Liverpool.

Llanelly Hospital.—Miss S. D. Richards has been appointed charge nurse. She was trained at the Fulham Infirmary, Hammersmith.

Southwark Infirmary, East Dulwich Grove, S.E.—Miss M. C. Fraser has been appointed sister. She was trained at the Royal Infirmary, Aberdeen, where she was afterwards staff nurse. She served in South Africa on the Army Nursing Service Reserve, and holds the L.O.S. certificate.

Weston-super-Mare Hospital. — Miss Helena Ada Elliot has been appointed matron. She was trained at the Royal Devon and Exeter Hospital, where she has since been sister and assistant matron.

### Echoes from the Outside World.

### Movements of Royalty.

On Saturday the King and Queen cruised about the Solent, the King on board the yacht *Meteor*, belonging to the German Emperor, and the Queen on board the *Osborne*. Divine service was held on Sunday morning on the deck of the yacht, the *Albert and Victoria*, at which their Majesties were present. The prayers were read by Commodore Sir Berkeley Milne, and the singing was accompanied by the band of the Royal Marine Artillery, who had been on board during the King's cruise. On Monday their Majesties, accompanied by the Prince of Wales and Princess Victoria, travelled to London, and received an enthusiastic greeting on their arrival at Victoria Station. After luncheon the King and Queen spent some time on the balcony of Buckingham Palace inspecting the progress which is being made with the national memorial to Queen Victoria. On Tuesday, at noon, they left St. Pancras for Sandringham, in order to attend the christening of the infant son of Prince and Princess Charles of Denmark, at which they acted as sponsors. The young Prince was baptised by the names Alexander Edward Christian Frederick. On Tuesday evening the King returned to London, and on Wednesday morning he left again for Marienbad, travelling as the Duke of Lancaster.

### The Pope's Coronation.

On Sunday morning Pope Pius X. was formally crowned in St. Peter's at Rome in presence of a vast crowd. The function was very elaborate, and occupied five hours, but most of the people were in the Basilica from 6 o'clock in the morning, three hours and a half before it commenced. The cortège which assembled in the Vatican consisted, first, of the Master of the Ceremonies, the procurators of the colleges, the preacher and confessor of the Pontifical household, and the procurators-general of the religious orders, attended by Swiss Guards. Then followed a group of footmen in red livery, and the chaplain carrying the tiara on a cushion, behind him being the chaplains in ordinary carrying the mitres. After these came a throng of ecclesiastical officials, the singers of the Pontifical chapel, the Greek clergy who read in Greek the Epistle and Gospel, and the penitentiaries of St. Peter wearing white chasubles. The mitred abbots were next in order, bishops, archbishops, patriarchs, and cardinals succeeding. Last of all came the chair, borne by *sediarii* in red livery, in which the Pope was seated. His Holiness was robed in all the vestments of the Pontifical Mass at the Chapel of St. Gregory, and was invested with the pallium on his arrival at the altar. On the termination of Mass, he was escorted to a high throne erected before the Altar of the Confession, and here the actual ceremony of coronation took place. Silver trumpets announced the conclusion, and finally the Pope, having recited the three prayers which follow that of coronation, gave his solemn benediction to the assembly.

### The New Governor-General of Australia.

Lord Northcote, whose appointment to the post of Governor-General of Australia has been approved by the King, is the second son of the first Earl of Iddesleigh—so long known in the country as Sir Stafford Northcote—and married in 1873, Alice, the adopted daughter and heiress of Lord Mount-Stephen. He sat in the House of Commons for Exeter from 1885 to 1900, when, after being Financial Secretary to the War Office, and Surveyor-General of Ordnance, he was appointed Governor of Bombay. It is understood that Queen Victoria, in approving his advance to the peerage, expressed a wish that the title should be associated with the family name in order to carry on the remembrance of his father's long association with the House of Commons.

### Electric Railway Disaster in Paris.

On Monday evening a terrible disaster occurred on the Paris Underground Electric Railway. According to the Paris correspondent of the *Times*, the motor of a train got out of order, and it would not proceed. The passengers, as well as those of another train following at a short interval, were directed to alight, and the second train then moved up to the first and began pushing it towards the terminus. The two trains were running at a considerable speed through a tunnel beyond the Couronnes Station, when there was a violent explosion. All the carriages caught fire, and employées had to fly for their lives. Another train full of passengers was brought to a standstill about 300 yards from the burning carriages. The occupants became panic-stricken, and on getting into the dark tunnel ran in different directions. The result was that between 80 and 90 persons were asphyxiated, or killed in other ways. At the instance of King Edward, the British Ambassador in Paris has offered to the French Government an expression of his Majesty's sorrow and deep sympathy.

### The Humbert Trial.

The story of Madame Humbert's alleged fortune is that while she was travelling in a train a gentleman was seized with a fit. She loosened his neckcloth, assisted him with brandy, and, according to a doctor whom she said was called, saved his life. This personage, it is asserted, she afterwards discovered to be an American millionaire, Henry Robert Crawford, who subsequently died at Nice, leaving her four millions sterling. To account for the non-possession of the money so left to her, Madame Humbert is accused of having invented two nephews of the deceased millionaire, whom she averred turned up and produced a new will causing such legal complications that neither she nor they could touch the property. The money was supposed to be locked in a safe; and the safe certainly existed. But when, after many years, the safe was opened, Madame Humbert having in the interval obtained thousands on expectations, it was found to contain only an empty jewel case, some bonds worth £200, a copper coin, and a button. The trial commenced on Saturday, and was disappointing to those who expected startling disclosures. Madame Humbert continually promised to make revelations calculated to shatter Society to its foundations, but only evaded replying to all the judge's questions. She wound up her opening improvisation by the words "We are the most honest people in all France." On Monday Madame Humbert made a partial confession. She said "The millions exist. The Crawfords exist. But the name itself does not exist. They bear another name." She also stated that she had given the name to her counsel, M. Labori, because she might die. On Tuesday M. Parmentier partially confirmed Madame Humbert's assertion that it was she who first suggested the opening of the safe.

### Death of Phil May.

On Saturday last the mortal remains of Mr. Phil May, the popular caricaturist, were laid to rest in Kensal Green Cemetery. Phil May, who was 39, was born at Wortley in Yorkshire. When he was 15 he obtained employment at the Spa Theatre, Scarborough, where for his services in costume-designing and making coloured character sketches for the bills, he was paid 12s. a week. Two years later he came to London, and his uncle, having shown him the sights of the capital, put him in a train for Leeds. But he got out of the train at the first stopping place and began his struggle for work. At times, for lack of money, he had to sleep under the carts in Covent Garden. However a cartoon which was accepted by a society paper proved the initial step to regular and fairly remunerative employment. From that period he did not look back, but his real artistic genius was not developed until he had visited Rome and Paris. He had for some years been recognised as a master of black and white.

### A Book and its Story.

### ELEVEN WITTY IRISH SKETCHES.*

Anyone who has read "Some Experiences of an Irish R.M." and other books by those clever authors E. Somerville and Martin Ross will know that laughter lurks between the covers of any book coming from them. Their latest one, "All on an Irish Shore," is infinitely diverting, and the curious corners in which the various characters find themselves, their mode of escape, and the ready repartee ever at hand in moments of difficulty, make very excellent reading. Many of the stories have a sporting turn, for every Irishman loves a horse; and dogs, too, are not forgotten, they hold their own in more than one sketch. It is impossible to convey in a passing notice the exhilarating atmosphere of the book. We will start on "The Dane's Breechin'." Someone—her name is left to our imagination—and Robert, were staying in a cottage in the country, lent by an aunt. It was presided over by Julia, an Irish maid, infinite in resource and of unswerving loyalty to her absent mistress. Someone, and Robert who is her brother, had made final arrangements of a minute nature for an expedition. Robert is a fisherman and Someone an artist. The sketching materials were strapped together, the fishing paraphernalia collected, a car ordered from the villiage public-house; nothing but the cold luncheon ordered remained to be added to the collection. But this depended on Julia. "In fact it would be difficult to mention anything at Wave-Crest Cottage that did not depend upon Julia. We who were but strangers and sojourners (the cottage with the beautiful name having been lent to us with Julia), felt that our very existence hung upon her clemency. Even courageous folks are afraid of other people's servants, and Robert and I were not courageous. Perhaps this was why Julia treated us with compassion, even with kindness, especially Robert.

"'Ah, poor Master Robert,' I've heard her say to a friend in the kitchen, who was fortunately hard of hearing, 'Ye wouldn't feel him in the house no more than a feather!'"

Everything being in train for the expedition, Someone went to the window to observe the weather, when her attention was diverted by the apparition of a covered car making for the modest entrance gates. Some description of this is necessary. "A covered car is peculiar to the South of Ireland; it resembles a two-wheeled waggonette with a windowless black box on the top of it. Its mouth is at the back, and it has the sinister quality of totally concealing its occupants until the irrevocable moment when it is turned and backed against your front door steps. For this moment my brother and I did not wait."

Retreat was the only course open. Having called to Julia in passing, "Say we're out—gone out for the day! We're going into the garden! "Sure ye needn't give yourself that much trouble," replied Julia affably, as she snatched a grimy cap off a nail. Nevertheless, in spite of the elasticity of Julia's conscience, the garden seemed safer."

Plunged in the dense thicket of the raspberry canes, the fugitives hid themselves and "the fulness of the situation began to sink into our souls." From their ambush they caught a glimpse through the drawing-room window of the visitors. With Aunt Dora's house had been taken on the goodwill of her acquaintance. In the party in the drawing room were recognised "the very flower and apex of the latter in the persons of the Dean of Glendad and Mrs. Doherty. Mrs. Doherty had brought her sister, Miss McEvoy, who was an elderly lady of the class usually described as 'being not all there.'" The drive of nine miles on a hot June day could not have been undertaken without the sustaining expectation of luncheon at the end of it. The knowledge that three mutton chops would hardly meet the expectation, communicated hurriedly to Robert, did not relieve the situation. "The consternation of his face, framed in the raspberry boughs, was a picture not to be lightly forgotten." At this moment Julia emerges from the kitchen, comes into the kitchen garden, her face wearing an expression of elaborate unconcern. "She picked a handful of parsley, her black eyes, questing for us among the bushes, met mine, and a glance more alive with conspiracy it has not been my lot to receive. She moved desultorily towards us, gathering green gooseberries in her apron. 'I told them the two of ye were out,' she murmured to the gooseberry bushes. 'They axed when would ye be back. I said you went to town on the early train and wouldn't be back till night.' Decidedly Julia's conscience could stand alone." Finally, a hasty flight over the garden wall, a hastier lunch at Coolahan's Inn, after which Robert, becoming restive, says he must at all hazards return to the dining-room for his fly book. Here, after secreting himself under the table, at the sound of footsteps, he remains until there is silence. Miss McEvoy having come into the room in search of biscuits, and, he imagines, having gone out again, he makes a bolt for it. His sister, in waiting, is confronted by the nightmare spectacle of her brother creeping on all fours through the open dining-room door, and then, crowning this already overloaded moment, there arose a series of yells from Miss McEvoy as bloodcurdling as they were inexcusable yet "something muffled by a Marie biscuit." Then the voice of the Dean is heard demanding "in a voice of cathedral-like severity, from the house at large," an explanation. "I gripped Robert's arm. The issues of life and death were now in Julia's hands. In reply to the question of 'Where is the man who was secreted under the dinner table?' Julia replies in honeyed tones, 'Sure that was the carpenter's boy, that came to quinch a rat hole.' 'But,' pursued the Dean raising his voice, 'I understand that he left the room on his hands and knees. He must have been drunk!' 'Not at all, your Reverence,' replied Julia, with almost compassionate superiority, 'sure that poor boy is the gentlest crayture that ever came into a house; I suppose 'tis what it was he was ashamed like when McEvoy commenced to screech, and faith he never stopped till he ran out of the house like a wild goose.'" One deception leads to another until there is a tangle of incident which ends in hibernian fashion. "An Irish Problem" is a police-court sketch; the defendant Sweeney is charged with sheep-worrying. The scene is extremely funny. There are family affairs, and various other matters introduced to influence the verdict, and a curious mixture of inconsistency and irrelevance marks the evidence. Finally the magistrate suggests that it would be as well to inquire into the value of the sheep. This ranged from one pound claimed by the plaintiff to sixpence by the defendant. "Her age swung like a pendulum between two years and fourteen, and in crowning proof of her general attractiveness it was stated that her own twin had been sold for fifteen shillings to the police at Dhulish 'ere last week.' Ten shillings is ultimately decided upon as compensation. The magistrate remarks 'Well that's all right, it's a funny thing but I notice that the defendant brought the exact sum into court with him.' 'I did! and I am able to bring more than it, thanks be to God!' said Sweeney fiercely, with all the offended pride of his race."

A note to Irish tourists and others "equipped with feeling hearts and the belief that two and two make four," is amusing. "In this country two and two are just as likely to make five, or three, or nothing at all."

* "All on an Irish Shore." By E. Œ. Somerville and Martin Ross. (Longmans. 6s.)

### Everybody's Opinion.

[Correspondence on all subjects is invited, but we cannot in any way be responsible for the opinions expressed by our correspondents. No communication can be entertained if the name and address of the correspondent are not given as a guarantee of good faith, but not necessarily for publication. All correspondents should write on one side of the paper only.]

### THE UNIFORM QUESTION AGAIN.

"A Cambridgeshire Nurse" writes: I appeal to your kindness to help all professional nurses in their efforts to prevent untrained persons, whether nursemaids or advertisers of patent medicines, from wearing our uniform. We have given years of hard work for the right to wear it, and should be only too proud to do so were it not for the fact that every other nursemaid we meet wears it also. Moreover, women who behave badly in uniform and bring discredit upon our calling are generally those who have no right to wear it. I am a nurse of 14 years' standing, and my many nursing friends are unanimous in wishing that something could be done to hinder women wearing uniform who are not professional nurses.

### THE TRAINING OF MIDWIVES.

"A Midwife and Nurse" writes: I am delighted with the idea of midwives returning to their hospital for re-examination and instruction, and I feel sure that it ought to be put into action. Every intelligent nurse will be only too glad to avail herself of the privilege. Six months' training is too great a rush, and no midwife's certificate should be complete without a course of gynæcology. Some time devoted to cookery and some time to washing would also be of great use. It is well to know how to do everything, and no one is as often in great straits as a maternity nurse. It is time to raise the standard of midwives, and matrons should be most strict about the women whom they take to train. I think it would be a good thing if nurses had to report themselves from time to time to their matron, and were obliged to keep a case book. This would not only encourage, but draw out the nurse's best efforts.

### NURSES OF THE SERVANT CLASS.

"General Trained" writes: There are many women who have been in domestic service who are intelligent and refined, possess a high sense of duty, and who, with proper training, prove capable and trustworthy nurses, and it is surely to their credit to endeavour to improve their position. With the existing large demand for nurses, it is impossible to attempt to exclude all but well-educated gentlewomen from the nursing ranks, although it is not wise on a limited staff to have nurses from widely different social classes. It is unfair to the better-class girl, and creates an uncomfortable position for her less well-bred colleague. The staffing of fever hospitals must always present many difficulties. One objection is that a slack season brings a reduction of staff; again, many fever hospitals are situated in very isolated and out-of-the-way places, especially those belonging to joint authorities. Many, again, with a parsimonious committee are considerably understaffed, and the work cannot be properly done. Perhaps we should get more satisfactory assistant fever nurses if fever training were not thought so little of, as it is by many. Young people go into a fever hospital for training, because they are too young for a general hospital; they do not value the instruction they receive, and often take little heed of it, because they have been taught to believe that general training is the only thing worth having. There are many women whom all the training procurable would never fit for a responsible post; they make good assistant fever nurses, and should always work under supervision. If such nurses would be content to stick to fever work, it would be better for themselves and for many others.

### Notes and Queries.

### REGULATIONS.

The Editor is always willing to answer in this column, without any fee, all reasonable questions, as soon as possible.

But the following rules must be carefully observed:—

1. Every communication must be accompanied by the name and address of the writer.
2. The question must always bear upon nursing, directly or indirectly.

If an answer is required by letter a fee of half-a-crown must be enclosed with the note containing the inquiry.

*Outdoor Hut.*

(199) Will you kindly tell me where I could buy a hut for a patient, who is undergoing the outdoor open-air treatment, to live in?—*F. P.*

Either from Messrs. Boulton and Paul, Limited, Norwich, or the Portable Building Company, Limited, Fleetwood.

*South Africa.*

(200) Will you kindly tell me how I can get into the hospitals in the mining districts in South Africa?—*H. R.*

For list of hospitals consult "Burdett's Hospitals and Charities," then write to the matrons and ask for vacancies.

*Receipt.*

(201) Will you kindly say if it is compulsory for a district nurse whose salary is paid monthly by cheque, to give a stamped receipt each month. My salary is £35 a year?—*H. M. G.*

A note acknowledging receipt of cheque is usually sufficient, but if required, you must use a stamp.

*Outfits for South Africa.*

(202) I am applying for a post in South Africa, and I should be glad if you could tell me where I could obtain any information as regards the outfit.—*E. W.*

The Secretary, the South African Expansion Emigration Committee, 47 Victoria Street, will doubtless be able to give you detailed information.

*L.O.S.*

(203) Will you kindly tell me the cheapest way for a nurse who is dependent on her earnings to get her L O.S.?—*K.A. S.*

The Clapham Maternity Hospital, Jeffrey's Road, Clapham, S.W., offers special terms to trained nurses. You have a District Nursing Association at Colchester, is there not a midwife attached to it who would prepare you privately for the examinations of the London Obstetrical Society? If so, write to the Secretary, 20 Hanover Square, W., for rules and particulars.

*Norland Institute.*

(204) Will you kindly give me the address of the Norland Institute?—*C. D. W.*

10 Pembridge Square, W.

*Massage.*

(205) Will you kindly tell me where I could learn massage in Liverpool?—*Nurse G.*

We have not the names of any public school for teaching massage in Liverpool, but doubtless the Secretary, the Incorporated Society of Trained Masseuses, 12 Buckingham Street, Strand, W.C., could give you a satisfactory recommendation.

*Registered Nurse.*

(206) Could you kindly tell me where I could register myself as a qualified nurse in Lancashire?—*J. W.*

There is no registration of nurses in England.

*Uniform.*

(207) Is an attendant trained only for mental work in a county asylum entitled to wear full nurse's uniform?—*B. H.*

Anyone who chooses is entitled to wear full-nurse's uniform.

*Books of Advice.*

(208) Kindly let me know of good books on "advice to a wife" and "advice to a mother"—*M. C.*

Chervasse's "Advice to a Wife" and "Advice to a Mother" are both good. You can get them through any bookseller, or from the Manager of the Scientific Press. The Manager would also be able to give you a list of the most recent publications on this and kindred subjects.

*Bexhill-on-Sea.*

(209) Will you kindly tell me if there is an institution for trained nurses at Bexhill-on-Sea, or a hospital?—*R. S.*

It is proposed to build a cottage hospital. At present there are only a Convalescent Home and two Sanatoria.

*Abroad.*

(210) Can you tell me the best way to find employment abroad, either in the Colonies or on the Continent? I am a fully certifcated obstetric nurse and midwife, holding a certificate for one year's general, and one year's surgical training.—*R. S.*

You might write to the Secretary, the Colonial Nursing Association, Imperial Institute, S.W.

*Maternity Fees.*

(211) If a maternity nurse is engaged for the 20th of one month, and is not required until the 15th of the next month, is she entitled to half fees for the time she is kept waiting? What is the general rule?—*Nurse D.*

Arrangements should be made at the time of the engagement. It is very customary to accept half fees.

*Margate.*

(212) Can you kindly tell me the addresses of the best convalescent homes for adults and children at Margate? I have looked in "Burdett," but can only find two mentioned there.—*D. U. P.*

We have not the names of any others.

*Album of Testimonials.*

(213) Will you kindly tell me if it is customary amongst nurses to keep albums in which patients or their friends may write testimonials?—*Greystoke.*

It is customary and businesslike to secure testimonials from the medical men under whom nurses have worked, and from patients, as such testimonials are necessary in order to gain future work.

*Nursing Home.*

(214) I have tried to get on the staff of several nursing homes in London but have been refused because I am a Roman Catholic.—*Nurse B.*

Why not advertise? The fact that you are a Roman Catholic would be a recommendation to matrons of some nursing homes.

*Holt-Ockley.*

(215) What is meant by the Holt-Ockley system of district nursing? and what are the advantages of being a Queen's Nurse?—*Nurse L.*

The Holt-Ockley is a system of employing women with a little knowledge of nursing to take charge of the sick poor and to help in the domestic work of the family. A Queen's Nurse, on the other hand, is a trained nurse who has had special training in district work. She belongs to Queen Victoria's Jubilee Institute for Nurses, and wears a badge indicating the fact. She visits the sick and fulfils all the duties to the patients under her care, but she is not responsible for attendance on any other member of the family.

*Hodgkin's Disease.*

(216) Will you kindly tell me what Hodgkin's disease is?—*M. S. P.*

Hodgkin's disease is a constitutional affection in which there is progressive enlargement of lymphatic glands with anæmia.

*Technical Term.*

(217) A Constant Reader would be glad to know what is the technical medical term for the operation of removing a nasal polypus.

There is no special technical term for the operation.

*Books.*

(218) Can you tell me what books would be required for pupil midwife at the Queen Charlotte's Lying-in Hospital?—*E. F.*

Write and ask the matron.

*Convalescent Homes for Nurses.*

(219) I shall be much obliged if you will inform me if there is any such institution as a convalescent home for nurses. I believe that nurses who belong to her Majesty the Queen's Guild have some such institution provided for them, but there are thousands of nurses who do not belong to this guild. Is there any home of this nature available for them in case of need? I shall be much obliged for any information you can afford as to this; and if there is no such institution, whether there is a want of the same?—*C. M. W.*

There are a few homes devoted to nurses exclusively, and in many they are received on moderate terms.

**Important Nursing Textbooks.**

"The Nursing Profession: How and where to Train." 2s. net; 2s. 4d. post free.

"A Handbook for Nurses." (New Edition). 5s. net; 5s. 4d. post free.

"The Human Body." 5s. post free.

"Ophthalmic Nursing." (New Edition). 3s. 6d. net; 3s. 10d. post free.

"Gynæcological Nursing." 1s. post free.

"Art of Feeding the Invalid." (Popular Edition). 1s. 6d. post free.

"Practical Hints on District Nursing." 1s. post free.

### For Reading to the Sick.

"CASTING ALL YOUR CARE UPON HIM."

O Lord, how happy should we be
If we could cast our care on Thee,
If we from self could rest;
And feel at heart that One above,
In perfect wisdom, perfect love,
Is working for the best.

*J. Anstice.*

He guides our feet, He guards our way,
His morning smiles bless all the day;
He spreads the evening veil, and keeps
The silent hours while Israel sleeps.

*I. Watts.*

It is a great point to learn the reality of what we often say; *i.e.*, that our cross is a daily one. There would be less depression, less fretfulness, less disheartenment, among us if we kept this more in mind. If one could always accept the small vexations and worries, the petty contradictions and annoyances caused by ourselves or by others, as what we call them conventionally—crosses, they would mould and discipline, instead of irritating and fretting us. Someone has said, that while we owe a large sum to God, He allows us to pay in small instalments by patience under these trifling trials. When eventide comes, we must go home, we elder children must go to our Father's house. Be patient, then—patient with others, patient with yourself.—*S. Lear.*

One great characteristic of holiness is never to be exacting—never to complain. Each complaint drags us down a degree in our upward course. If you would discern in whom God's spirit dwells, watch that person, and notice whether you ever hear him murmur.—*Gold Dust.*

Little things come daily, hourly, within our reach, and they are not less calculated to set forward our growth in holiness, than are the greater occasions which occur but rarely. Moreover, fidelity in trifles, and an earnest seeking to please God in little matters, is a test of real devotion and love. Let your aim be to please our dear Lord perfectly in little things, and to attain a spirit of childlike simplicity and dependence. In proportion as self-love and self-confidence are weakened, and our will bowed to that of God, so will hindrances disappear, the internal troubles and contests which harassed the soul vanish, and it will be filled with peace and tranquillity.—*Jean Nicolas Grou.*

A soul occupied with great ideas best performs small duties; the divinest views of life penetrate most clearly into the meanest emergencies; so far from petty principles being best proportioned to petty trials, a heavenly spirit taking up its abode with us can alone sustain well the daily toils, and tranquilly pass the humiliations of our condition. —*J. Martineau.*

To have, each day, the thing I wish,
Lord, that seems best to me;
But not to have the thing I wish,
Lord, that seems best to Thee.
Most truly, then, Thy will is done,
When mine, O Lord, is crossed;
'Tis good to see my plans o'erthrown,
My ways in Thine all lost.

*H. Bonar.*

